# The mechanisms influencing AI teaching readiness among preservice STEM teachers: an empirical analysis based on PLS-SEM and fsQCA

**DOI:** 10.3389/fpsyg.2026.1868148

**Published:** 2026-06-26

**Authors:** Hongbo Zhou, Chen Chen, Yanlong Xie

**Affiliations:** 1 College of Teacher Education, Ningxia University, Yinchuan, China; 2 School of Mathematics and Computer Science, Ningxia Normal University, Guyuan, China

**Keywords:** AI teaching readiness, fsQCA, PLS-SEM, preservice STEM teachers, technology acceptance model, technology readiness

## Abstract

**Introduction:**

With the rapid integration of artificial intelligence (AI) technologies into basic education, preservice teachers’ AI teaching readiness has become an important factor influencing future AI-supported teaching practices and instructional innovation. Drawing on technology readiness (TR), the technology acceptance model (TAM), and the stimulus–organism–response (SOR) framework, this study developed a mechanism model to explain AI teaching readiness among preservice STEM teachers.

**Methods:**

This study empirically examined the proposed model using partial least squares structural equation modeling (PLS-SEM) and fuzzy-set qualitative comparative analysis (fsQCA). In the model, school support was conceptualized as an external stimulus; technology readiness traits, including optimism, innovativeness, discomfort, and insecurity, together with technology-related cognitive appraisals, including perceived usefulness and perceived ease of use, were conceptualized as internal organismic states; and AI teaching readiness was conceptualized as the response.

**Results:**

The findings showed that perceived usefulness and perceived ease of use were key factors driving preservice STEM teachers’ AI teaching readiness. School support influenced AI teaching readiness through multiple chain-mediating paths involving technology readiness traits and technology-related cognitive appraisals. The PLS-SEM results indicated that the effects of school support on innovativeness and insecurity were not significant. Further, the fsQCA results revealed that, in some high-readiness configurations, school support worked jointly with innovativeness, insecurity, and technology-related cognitive appraisals, while high innovativeness could also compensate, to some extent, for insufficient school support.

**Discussion:**

The cross-analysis of PLS-SEM and fsQCA revealed both linear pathways and configurational effects in the formation of AI teaching readiness, suggesting that it is not driven by a single factor but by combinations of multiple conditions. This study enriches the application of TR, TAM, and SOR in the context of AI educational technology adoption and provides practical implications for teacher education institutions and school administrators seeking to enhance preservice teachers’ AI teaching readiness.

## 1 Introduction

With the rapid development of artificial intelligence (AI) technologies, AI is increasingly becoming an important force driving digital transformation and instructional innovation in education (Tan et al., 2024; Belkina et al., 2025). Preservice STEM teachers are at a critical stage in the formation of their professional knowledge, teaching competence, and technology-related beliefs. Their perceptions, attitudes, and experiences with AI technologies may influence how they integrate technology into future teaching practices (Reyes-Rojas et al., 2025; Sun et al., 2024; Guan et al., 2024; Runge et al., 2025). Meanwhile, STEM education is characterized by interdisciplinarity, practice orientation, and technology intensity, which closely align with AI-supported instructional scenarios such as data analysis, intelligent simulation, problem solving, and personalized learning (Leon et al., 2025; Li et al., 2025; Sun D. et al., 2025; Cooper, 2023; Otto et al., 2025; Lee et al., 2025; Feldman-Maggor et al., 2024; Avci et al., 2025). Therefore, preservice STEM teachers need not only general technology integration competence but also the awareness, beliefs, and readiness to apply AI tools appropriately in STEM teaching contexts. Exploring the mechanisms underlying preservice STEM teachers’ AI teaching readiness is therefore important for teacher education to respond to instructional transformation in the intelligent era.

Against the background of the continued expansion of AI applications in education, preservice STEM teachers’ acceptance of AI technologies, intention to use AI, and integration of AI into teaching have gradually become important topics in empirical research. Existing studies indicate that teachers’ AI acceptance and instructional integration involve a complex process shaped by technology-related cognition, competence beliefs, pedagogical knowledge bases, and contextual support (Beege et al., 2024; Sun et al., 2024; Ramnarain et al., 2024; Wang X. et al., 2023; Zhang et al., 2023; Hsu et al., 2025; Zhao et al., 2024). For example, Beege et al. (2024), focusing on secondary STEM teachers in Germany, found that teachers’ perceived competence and perceived benefits positively influenced their use of AI tools and future intention to use them, suggesting that STEM teachers’ willingness to adopt AI tools is closely associated with their competence perceptions and value judgments. Sun et al. (2024) examined preservice STEM teachers’ willingness to integrate AI into STEM teaching and its influencing factors. Their findings showed that TPACK, perceived usefulness, perceived ease of use, and self-efficacy all affected preservice STEM teachers’ intention to integrate AI, indicating that their AI teaching readiness is influenced not only by their technological knowledge base but also by technology-related cognitive appraisals and self-beliefs. Together, these studies reveal the factors influencing teachers’ adoption and integration of AI technologies from different perspectives and provide important empirical foundations for understanding preservice STEM teachers’ AI teaching readiness.

However, existing research has primarily focused on AI integration intention or technology adoption behavior, while systematic explanations of the mechanisms underlying preservice STEM teachers’ AI teaching readiness remain limited. In particular, few studies have incorporated external support, technology readiness traits, and technology-related cognitive appraisals into a unified theoretical framework to examine how these factors jointly shape preservice STEM teachers’ AI teaching readiness. Given that the formation of AI teaching readiness involves multidimensional interactions and complex causal relationships, relying solely on a linear analytical framework may be insufficient to reveal differences in readiness resulting from different combinations of factors. Therefore, a more integrated theoretical perspective and a multi-method research strategy are needed to systematically explain the formation mechanism of AI teaching readiness among preservice STEM teachers.

Based on this rationale, this study draws on the stimulus–organism–response (SOR) theory to construct a mechanism model of AI teaching readiness among preservice STEM teachers. According to SOR theory, external stimuli, namely school support, influence individuals’ internal psychological states, including TR traits and TAM-based cognitive appraisals, which in turn shape their final readiness response. This framework helps integrate external environmental factors with teachers’ internal psychological mechanisms, thereby providing a systematic pathway for understanding AI teaching readiness. Methodologically, this study adopts a dual-method strategy combining partial least squares structural equation modeling (PLS-SEM) and fuzzy-set qualitative comparative analysis (fsQCA). PLS-SEM is used to examine the linear relationships among the conditional variables, whereas fsQCA is used to identify multiple equifinal pathways through which different combinations of conditions lead to high readiness. These two methods complement each other and together provide a more comprehensive understanding of the complex formation mechanism of AI teaching readiness.

Taken together, this study addresses the following four research questions:

1. To what extent can the SOR-based model explain AI teaching readiness?
2. What are the key factors influencing AI teaching readiness among preservice STEM teachers?
3. How do environmental factors, affective experiences, and individual cognition jointly shape AI teaching readiness?
4. Which configurations of conditions lead to different pathways of high AI teaching readiness?

## 2 Literature review and research hypotheses

### 2.1 Application of AI in STEM education

In recent years, artificial intelligence (AI) has developed rapidly across basic and higher education systems and has gradually become an important technological force driving the transformation of STEM education. Because STEM courses often involve abstract concepts, complex problem situations, and the cultivation of higher-order thinking, traditional teaching approaches still have certain limitations in providing personalized support, dynamic feedback, and contextualized inquiry (Tan et al., 2022; Villegas-Ch et al., 2025). The integration of AI provides new pathways for addressing these instructional challenges (Leon et al., 2025; Yu et al., 2025a; Jia et al., 2023). Existing studies generally suggest that AI has great potential to reshape STEM teaching models, enhance learning support systems, and promote personalized learning (Wu et al., 2023; Lin et al., 2022). This potential is reflected not only in its support for the learning process but also in the continuous optimization of instructional interaction and teaching decision-making. Accordingly, the value of AI applications in STEM education is mainly reflected in the following aspects.

First, AI can provide highly personalized learning support. According to Xu and Ouyang (2022), AI tools can generate differentiated learning pathways based on students’ learning progress and performance data, provide real-time feedback, and help students overcome difficulties in understanding scientific concepts, thereby making the learning process more responsive to individual differences. Such adaptive learning not only enhances learning motivation but also helps improve learning outcomes, which is particularly important for students who experience difficulties in STEM learning. Second, AI promotes the development of interactive and immersive learning experiences. Studies have shown that AI-driven virtual laboratories and intelligent simulation environments enable students to engage in scientific inquiry activities under safe and low-cost conditions, thereby improving their operational experience and problem-solving ability (Klami et al., 2024; Sajadieh and Noh, 2025). In addition, AI-generated visual models, automated feedback, and real-time data analysis make it easier for students to understand complex mathematical models, chemical reactions, or physical phenomena. These tools not only enhance the perceptibility of abstract content but also strengthen interaction and immersion in the learning process (Awang et al., 2025; Ma, 2025; Jang and Choi, 2024). Third, AI also plays an important role in instructional decision-making and learning analytics. With the support of learning data analysis and intelligent algorithms, teachers can more accurately identify students’ learning weaknesses, predict potential learning risks, and obtain decision support for monitoring learning progress, automatically grading assignments, and analyzing classroom interactions (Xu, 2025). These functions help reduce teachers’ repetitive workload, allowing them to devote more attention to instructional design, learning intervention, and individualized guidance.

However, the application of AI in STEM education is not without challenges. Hazzan-Bishara et al. (2025) noted that teachers may face technology anxiety, insufficient experience, and doubts about the ease of use and usefulness of AI tools when adopting them. In addition, inadequate organizational support, resource provision, and training mechanisms may further constrain the broader implementation of AI in STEM education. This suggests that the effective integration of AI technologies into STEM education depends not only on the functional advantages of the technologies themselves but also on the synergy among teachers’ technology readiness, cognitive appraisals, and external support conditions.

### 2.2 AI teaching readiness as an extended outcome of TAM

AI teaching readiness refers to teachers’ overall preparedness to understand, evaluate, and integrate AI technologies in teaching contexts. It reflects their cognition, competence, attitudes, and behavioral tendencies toward future AI-enabled teaching practices (Wang X. et al., 2023; Wang C. et al., 2023; Reyes-Rojas et al., 2025). Compared with general technology use intention, AI teaching readiness focuses not only on whether individuals are willing to use AI tools but also on whether they are prepared to apply AI to instructional design, classroom implementation, learning support, and teaching assessment (Guan et al., 2024; Reyes-Rojas et al., 2025). For preservice STEM teachers, such readiness further involves whether they can connect AI technologies with STEM teaching scenarios such as data analysis, intelligent simulation, problem solving, and personalized learning.

Traditional TAM usually regards use intention or actual use behavior as the outcome variable (Davis, 1989; Venkatesh and Davis, 2000). However, in teacher education contexts, the key issue facing preservice teachers is not merely whether they are willing to use AI, but whether they are ready to integrate AI into their future teaching practices. Therefore, AI teaching readiness is conceptually broader than use intention. It includes not only individuals’ willingness to use AI but also their judgments about the cognitive, competence-related, and practical preparedness required for AI integration in teaching.

From the logic of TAM, perceived usefulness and perceived ease of use, respectively, reflect individuals’ cognitive judgments of a technology’s value and operational feasibility (Davis, 1989). For preservice teachers, a higher level of AI teaching readiness is more likely to develop only when they perceive AI technologies as capable of enhancing instructional design, classroom implementation, and learning support, and as understandable, operable, and integrable into teaching practice (Lucas et al., 2025). Therefore, this study regards AI teaching readiness as an extension of the outcome logic of TAM in the teacher education context, aiming to explain the mechanism through which preservice STEM teachers move from “accepting AI technologies” to “being ready to apply AI in teaching.”

### 2.3 Stimulus-organism-response framework

The stimulus-organism-response (SOR) theory was proposed by Mehrabian and Russell (1974) to explain how individuals form behavioral responses to external situational stimuli through internal psychological and cognitive processing. According to this theory, individual behavior is not a direct result of external stimuli; rather, it emerges after such stimuli are transformed through internal states such as cognition, emotion, and perception. Its basic logic can be expressed as Stimulus → Organism → Response. In educational technology research, external conditions such as organizational support, technological environments, training resources, and learning support are often regarded as important stimuli that influence individuals’ technology-related cognition, attitudes, and use behaviors (Dahri et al., 2025; Ning et al., 2025; Jiang and Sun, 2025; Luckin et al., 2022).

In this study, school support is regarded as the external stimulus, reflected in the resources, training opportunities, technological environment, and emotional support provided by the school. Technology readiness traits and technology-related cognitive appraisals are regarded as organismic states, respectively reflecting preservice STEM teachers’ psychological tendencies toward AI technologies and their cognitive judgments of the pedagogical value and perceived difficulty of using AI. AI teaching readiness is regarded as the final response, reflecting their preparedness to integrate AI technologies into future STEM teaching practices. Thus, the SOR theory provides an overarching explanatory framework for integrating external support, individual psychological traits, technology-related cognitive appraisals, and AI teaching readiness in this study.

### 2.4 Technology readiness, technology acceptance model, and research hypotheses

In the SOR theory, the “organism” refers to individuals’ internal psychological states, cognitive appraisals, and affective responses formed under the influence of external stimuli. It serves as an important mediating mechanism through which external environmental factors influence behavioral outcomes. For preservice STEM teachers, school support, as an external stimulus, does not simply or directly translate into AI teaching readiness. Instead, it needs to be processed through individuals’ internal psychological tendencies and cognitive judgments. Therefore, this study introduces technology readiness theory and the technology acceptance model to characterize preservice STEM teachers’ organismic states from two dimensions: technology-related psychological tendencies and technology-related cognitive appraisals. This approach further explains the formation mechanism of their AI teaching readiness.

Technology readiness (TR), proposed by Parasuraman (2000), is used to measure individuals’ psychological tendencies and preparedness when facing new technologies. It consists of four dimensions: optimism, innovativeness, discomfort, and insecurity. Specifically, optimism refers to the tendency of individuals, in this study preservice STEM teachers, to believe that AI technologies can improve teaching efficiency, enrich instructional resources, and enhance teaching effectiveness. Innovativeness refers to individuals’ willingness to actively try new tools and explore the potential applications of AI technologies in instructional design, classroom interaction, and learning support. Together, these two dimensions constitute the positive psychological traits of technology readiness. By contrast, discomfort refers to a psychological state in which individuals feel a lack of control, high operational pressure, or insufficient competence when using technology. Insecurity refers to individuals’ doubts and concerns regarding the reliability, safety, and pedagogical appropriateness of technology. Together, these two dimensions constitute the negative psychological traits of technology readiness.

The technology acceptance model (TAM), proposed by Davis (1989), posits that perceived usefulness and perceived ease of use are important cognitive factors influencing individuals’ technology acceptance. Perceived usefulness refers to the extent to which individuals believe that a technology can improve their performance in teaching tasks, whereas perceived ease of use refers to the extent to which individuals believe that a technology is easy to learn and use. Lin et al. (2007) further integrated TR and TAM and proposed the technology readiness and acceptance model (TRAM), emphasizing that individuals’ technology readiness influences their judgments of a technology’s usefulness and ease of use, which in turn affects technology acceptance outcomes.

First, according to SOR theory, school support, as an external stimulus, can influence preservice teachers’ internal psychological states through resource provision, training opportunities, technological environments, and emotional support. Previous studies have shown that technology support, training resources, and practice opportunities at the school or organizational level can enhance teachers’ trust in AI technologies, positive attitudes, and confidence in use, while also helping reduce their anxiety and pressure when facing new technologies (Luckin et al., 2022; Nazaretsky et al., 2022; Hopcan et al., 2023). In AI teaching contexts, when schools provide sufficient hardware and software conditions, curriculum resources, technical guidance, and practice opportunities, preservice STEM teachers are more likely to develop positive expectations toward AI technologies and a willingness to actively explore their use, while reducing the psychological burden caused by technological complexity and uncertainty. Therefore, this study proposes the following hypotheses:

*H12*: School support positively affects optimism.

*H13*: School support positively affects innovativeness.

*H14*: School support negatively affects discomfort.

*H15*: School support negatively affects insecurity.

Second, within the TRAM framework, technology readiness is regarded as an important antecedent psychological condition influencing technology acceptance cognition. Previous studies related to TRAM have shown that positive technology readiness tends to enhance individuals’ perceived usefulness and perceived ease of use, whereas negative technology readiness may weaken their technology-related cognitive appraisals (Lin et al., 2007; Jin, 2019; Anh et al., 2024). In AI-supported teaching contexts, preservice STEM teachers with higher levels of optimism and innovativeness are more likely to regard AI as an effective tool for improving teaching efficiency, enriching instructional resources, and supporting personalized learning, thereby developing higher perceived usefulness and perceived ease of use. Conversely, higher levels of discomfort and insecurity may lead them to form negative judgments about the operational difficulty, reliability, and pedagogical value of AI tools, thereby weakening their perceptions of the usefulness and ease of use of AI technologies. Therefore, this study proposes the following hypotheses:

H4: Optimism positively affects perceived ease of use.

*H5*: Optimism positively affects perceived usefulness.

*H6*: Innovativeness positively affects perceived ease of use.

*H7*: Innovativeness positively affects perceived usefulness.

*H8*: Discomfort negatively affects perceived ease of use.

*H9*: Discomfort negatively affects perceived usefulness.

*H10*: Insecurity negatively affects perceived ease of use.

*H11*: Insecurity negatively affects perceived usefulness.

Finally, TAM suggests that perceived ease of use not only directly influences individuals’ technology acceptance outcomes but also exerts an indirect effect by enhancing perceived usefulness (Davis, 1989; Venkatesh and Davis, 2000). If preservice STEM teachers perceive AI tools as easy to learn, operate, and integrate into teaching practice, they are more likely to recognize the value of AI in instructional design, resource generation, learning analytics, and classroom support. Previous studies on AI tool acceptance have shown that perceived ease of use significantly promotes perceived usefulness, and that both perceived usefulness and perceived ease of use are important factors influencing technology acceptance and application readiness (Davis, 1989; Venkatesh and Davis, 2000; Lv et al., 2025; Xia et al., 2025). Therefore, in this study, if preservice STEM teachers perceive AI technologies as both useful for improving teaching task performance and easy to learn and use, their AI teaching readiness is also likely to increase. Based on this reasoning, this study proposes the following hypotheses:

*H3*: Perceived ease of use positively affects perceived usefulness.

*H2*: Perceived usefulness positively affects AI teaching readiness.

*H1*: Perceived ease of use positively affects AI teaching readiness.

Taken together, based on the SOR framework, this study conceptualizes school support as the external stimulus, technology readiness and TAM-based cognitive variables as organismic psychological–cognitive states, and AI teaching readiness as the response. This forms a dynamic influence chain of “school support → technology readiness → technology acceptance cognition → AI teaching readiness.” Specifically, TAM can explain preservice teachers’ cognitive judgments of the usefulness and ease of use of AI technologies, but it is less able to reveal the technology-related psychological differences and school environmental factors that precede the formation of these cognitions. TR can explain individuals’ psychological preparedness when facing new technologies, but it does not sufficiently explain how these psychological tendencies are further transformed into specific technology-related cognitive appraisals. SOR, in turn, provides an overarching logic for linking external support, technology-related psychology, technology-related cognition, and readiness outcomes. Therefore, this integrated framework not only helps explain the formation process of AI teaching readiness among preservice STEM teachers but also provides a theoretical foundation for subsequently examining both linear path relationships and complex configurational relationships among the variables.

**Figure 1:**
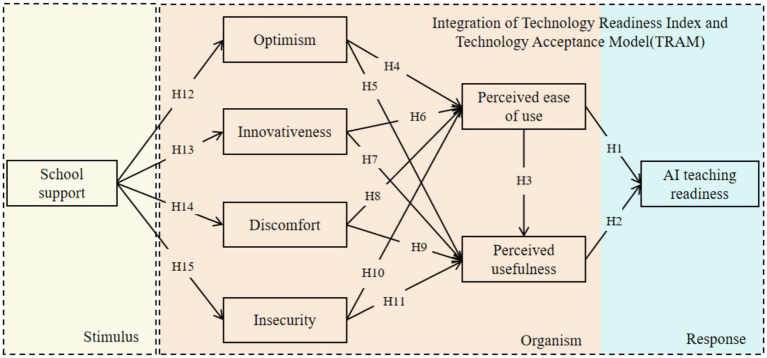
Proposed hypothesized model of AI teaching readiness among preservice STEM teachers.

Accordingly, this study proposes a hypothesized model of AI teaching readiness among preservice STEM teachers, as shown in Figure 1.

## 3 Research methodology

### 3.1 Measurement

To ensure the scientific rigor, reliability, and validity of the measurement instrument, the measurement items for each latent variable in this study were adapted from well-established scales and were appropriately contextualized to fit the research setting. Specifically, the scale for school support was adapted from Teo (2011) and Abbad (2021). The scales for optimism, innovativeness, discomfort, and insecurity were adapted from the technology readiness scale developed by Parasuraman and Colby (2014). The scales for perceived usefulness and perceived ease of use were adapted from Venkatesh and Davis (2000). The scale for AI teaching readiness was developed with reference to the measurement items related to AI use/learning behavioral intention in Wang et al. (2024) and Chai et al. (2021). School support consisted of five items, while all other variables were measured using four items. All items were rated on a five-point Likert scale ranging from “strongly disagree” (1) to “strongly agree” (5).

To enhance content validity, three experts in education and AI applications in education were invited to review and revise the questionnaire items, ensuring conceptual coverage and wording accuracy. Meanwhile, a translation–back-translation procedure was adopted to examine semantic consistency between the original English scales and the Chinese version of the questionnaire (Jin and Weber, 2013). Before the formal survey, 76 pilot questionnaires were distributed to preservice STEM teachers to examine item comprehensibility, discrimination, and internal consistency. Based on the pilot results, the wording of the questionnaire was further refined.

### 3.2 Data collection and sampling

The questionnaire used in this study consisted of three parts. The first part introduced the research background, explained the role of AI teaching readiness in preservice STEM education, and informed respondents of the principles of data confidentiality and voluntary participation. The second part measured the core research constructs related to AI teaching readiness among preservice STEM teachers. The third part collected demographic information, including gender, age, grade level, and teaching subject.

This study adopted convenience sampling, a type of non-probability sampling, to collect data. The researchers distributed paper-based questionnaires during class breaks to preservice teachers majoring in four teacher education programs—mathematics and applied mathematics, physics, computer science and technology, and chemistry—at two universities in western China.

To ensure data quality, this study followed the data screening procedure used by Wang C. et al. (2023) and established three questionnaire screening criteria. First, based on the pilot test results, the normal completion time of the questionnaire was approximately 3 to 6 min; therefore, responses completed in less than 2 min were regarded as invalid. Second, a reverse-coded item was included in the questionnaire, and responses from participants who failed to answer it correctly were removed. Finally, questionnaires with identical responses across all items were also excluded. In structural equation modeling research, a ratio of 10:1 between sample size and the number of estimated parameters or observed variables is often regarded as a relatively conservative minimum standard (Bentler and Chou, 1987; Hair et al., 2010). This study included 33 observed variables; therefore, the minimum required sample size was 330. In early December 2025, a total of 633 participants completed the questionnaire. After data cleaning based on the above screening criteria, 550 valid responses were retained, exceeding the minimum sample size requirement and thereby ensuring the robustness of the subsequent statistical analyses (Hair et al., 2016).

The demographic information of the final sample is presented in Table 1. Regarding the frequency of AI tool use, the proportions of respondents reporting use from “1–2 times per week” to “daily use” each exceeded 20%, indicating that the sample as a whole had a certain level of experience with AI tools.

**Table 1:** Demographics of respondents (*n* = 550).

Demographics	Classification	Number	Proportion (%)
Gender	Male	216	39.3
	Female	334	60.7
Age	Below 22 years	375	68.2
	23–25 years	102	18.5
	Above 26 years	73	13.3
Grade	Sophomore	87	15.8
	Junior	123	22.4
	Senior	175	31.8
	Postgraduate	165	30.0
Major	Mathematics and applied mathematics	173	31.5
	Physics	130	23.6
	Computer science and technology	152	27.6
	Chemistry	95	17.3
Frequency	Seldom use	41	7.5
	1–2 times each month	67	12.2
	1–2 times each week	152	27.6
	3–4 times each week	161	29.3
	Almost every day	129	23.4

### 3.3 Common method variance

This study used a single-source data collection approach, as all observed variables were measured through self-reported responses from the same group of participants. Therefore, there may be a potential risk of common method variance (CMV). To examine this issue, this study employed two assessment methods. First, following the recommendation of Podsakoff et al. (2003), Harman’s single-factor test was conducted as a preliminary assessment. The results showed that the first factor in the unrotated factor analysis explained 31.98% of the total variance, which was below the critical threshold of 50% (Sarstedt et al., 2014). This indicates that no single factor accounted for the majority of the variance and that CMV was not a serious concern.

Second, this study further applied the marker variable technique proposed by Lindell and Whitney (2001). Four items measuring “perceptions of the consequences of being late for work” were selected as the marker variable, as this construct was theoretically unrelated to the main variables of this study. The results showed that the correlations between the marker variable and the main research variables were low and non-significant, indicating that common method variance did not exert an obvious systematic influence on the relationships among the variables. Taken together, the results of these two tests suggest that no serious common method variance problem was detected in this study.

## 4 Data analysis and results

This study used SmartPLS 4.0 for data processing and analysis, with partial least squares structural equation modeling (PLS-SEM) employed as the primary statistical method. PLS-SEM was selected because the data in this study were non-normally distributed, and this method has notable advantages in handling non-normal data. In addition, PLS-SEM can maintain relatively high statistical power when analyzing complex models with multiple latent variables (Hair et al., 2016; Yu et al., 2025b).

### 4.1 Symmetric analysis

#### 4.1.1 Measurement model

To assess the quality of the measurement model, this study examined the reliability and validity of each construct. The internal consistency reliability of the constructs was mainly evaluated using Cronbach’s alpha and composite reliability (CR). Meanwhile, the reliability of individual measurement items was assessed based on their outer loadings. Convergent validity was examined using the average variance extracted (AVE), which reflects the average proportion of variance in the measurement indicators explained by the latent variable and serves as an important criterion for determining whether a construct has adequate convergent validity. According to the recommendations of Hair et al. (2019b, 2021), the measurement model should meet the following criteria: CR values should be greater than 0.70, AVE values should be greater than 0.50, Cronbach’s alpha values should exceed 0.70, and the factor loadings of all measurement items should exceed 0.70. The results of this study showed that all constructs met the above threshold criteria, indicating that the measurement model had high reliability and good convergent validity (Table 2). In addition, this study used the outer variance inflation factor (outer VIF) to examine indicator-level collinearity. As shown in Table 2, the outer VIF values ranged from 1.547 to 3.757, all below the recommended threshold of 5.0, indicating that there was no serious collinearity problem among the measurement indicators (Hair et al., 2019b).

**Table 2:** Measurement model assessment.

Construct	Items	Outer loading	Cronbach’s alpha	CR	AVE	Outer VIF
School support	SS1	0.839	0.907	0.930	0.728	2.274
	SS2	0.859				2.488
	SS3	0.849				2.406
	SS4	0.865				2.529
	SS5	0.854				2.454
Optimism	OPT1	0.886	0.903	0.932	0.775	2.686
	OPT2	0.869				2.430
	OPT3	0.874				2.538
	OPT4	0.893				2.811
Innovativeness	INN1	0.846	0.879	0.917	0.734	2.230
	INN2	0.837				2.124
	INN3	0.885				2.868
	INN4	0.858				2.575
Discomfort	DIS1	0.864	0.900	0.930	0.770	2.399
	DIS2	0.873				2.515
	DIS3	0.883				2.601
	DIS4	0.890				2.664
Insecurity	INS1	0.868	0.882	0.919	0.739	2.323
	INS2	0.856				2.240
	INS3	0.863				2.360
	INS4	0.850				2.077
Perceived ease of use	PEU1	0.835	0.815	0.878	0.644	1.817
	PEU2	0.805				1.706
	PEU3	0.811				1.710
	PEU4	0.756				1.547
Perceived usefulness	PU1	0.895	0.856	0.903	0.699	2.501
	PU2	0.824				1.925
	PU3	0.822				1.921
	PU4	0.800				1.797
AI teaching readiness	AITR1	0.912	0.937	0.955	0.841	3.488
	AITR2	0.922				3.757
	AITR3	0.915				3.640
	AITR4	0.919				3.678

Discriminant validity is an important criterion for evaluating the quality of the measurement model, as it assesses whether different latent constructs are sufficiently distinct from one another (Xia et al., 2024). This study evaluated discriminant validity using two methods. First, according to the criterion proposed by Fornell and Larcker (1981), discriminant validity is established when the square root of a construct’s AVE is greater than the absolute values of its correlations with other constructs. As shown in Table 3, all constructs in this study met this criterion. Second, this study further examined discriminant validity using the heterotrait–monotrait ratio (HTMT). The results showed that the highest HTMT value in this study was 0.759, which was below the strict threshold of 0.85 recommended by Hair et al. (2021). Taken together, the results of these two tests indicate that the measurement model in this study demonstrated good discriminant validity.

**Table 3:** Discriminant validity.

Construct	AITR	DIS	INN	INS	OPT	PEU	PU	SS
Fornell–Larcker criterion								
AI teaching readiness (AITR)	**0.917**							
Discomfort (DIS)	−0.341	**0.877**						
Innovativeness (INN)	0.419	−0.021	**0.857**					
Insecurity (INS)	0.107	0.105	0.252	**0.859**				
Optimism (OPT)	0.489	−0.256	0.024	0.203	**0.881**			
Perceived ease of use (PEU)	0.668	−0.339	0.399	0.182	0.450	**0.802**		
Perceived usefulness (PU)	0.659	−0.282	0.410	0.092	0.532	0.562	**0.836**	
School support (SS)	0.409	−0.485	0.075	−0.074	0.515	0.367	0.389	**0.853**
Heterotrait-monotrait ratio								
AI teaching readiness (AITR)								
Discomfort (DIS)	0.371							
Innovativeness (INN)	0.462	0.033						
Insecurity (INS)	0.117	0.119	0.285					
Optimism (OPT)	0.532	0.283	0.051	0.227				
Perceived ease of use (PEU)	0.759	0.398	0.471	0.216	0.520			
Perceived usefulness (PU)	0.729	0.319	0.471	0.106	0.601	0.665		
School support (SS)	0.443	0.536	0.083	0.082	0.569	0.426	0.439	

Values on diagonal, which are bold, are the square root of AVE. HTMT <0.85 (Hair et al., 2021).

#### 4.1.2 Structural model

After completing the assessment of the measurement model, this study further evaluated the structural model. To enhance the reliability and robustness of the structural model results, this study assessed the model following the analytical procedures proposed by Maroufkhani et al. (2022), including construct collinearity in the structural model, the significance of path relationships, the coefficient of determination (*R^2^*), effect size (*f^2^*), and predictive relevance (*Q^2^*). First, construct collinearity in the structural model was examined. The results showed that the VIF values ranged from 1.000 to 1.682 (Table 4), all of which were below the threshold of 5.0 recommended by Hair et al. (2019a). This indicates that there was no serious construct collinearity problem in the structural model.

**Table 4:** Results of structural model assessment.

H	Relationship	*β*	Std. dev.	*t*-value	*f^2^*	Confidence interval	VIF	*p-value*	Supported
H1	PEU → AITR	0.435	0.033	13.308	0.297	[0.368, 0.496]	1.461	< 0.001	Yes
H2	PU → AITR	0.414	0.031	13.435	0.269	[0.354, 0.476]	1.461	< 0.001	Yes
H3	PEU → PU	0.233	0.039	6.009	0.065	[0.156, 0.308]	1.682	< 0.001	Yes
H4	OPT → PEU	0.372	0.034	10.820	0.205	[0.303, 0.438]	1.137	< 0.001	Yes
H5	OPT → PU	0.422	0.033	12.718	0.262	[0.356, 0.486]	1.370	< 0.001	Yes
H6	INN → PEU	0.376	0.032	11.636	0.222	[0.312, 0.439]	1.073	< 0.001	Yes
H7	INN → PU	0.333	0.033	10.003	0.171	[0.270, 0.398]	1.310	< 0.001	Yes
H8	DIS → PEU	−0.240	0.034	6.960	0.088	[−0.307, −0.172]	1.104	< 0.001	Yes
H9	DIS → PU	−0.077	0.033	2.303	0.010	[−0.142, −0.014]	1.201	0.021	Yes
H10	INS → PEU	0.037	0.034	1.076	0.002	[−0.030, 0.105]	1.149	0.282	No
H11	INS → PU	−0.112	0.032	3.477	0.022	[−0.175, −0.047]	1.151	< 0.001	Yes
H12	SS → OPT	0.515	0.033	15.792	0.361	[0.448, 0.575]	1.000	< 0.001	Yes
H13	SS → INN	0.075	0.042	1.762	0.006	[−0.008, 0.159]	1.000	0.078	No
H14	SS → DIS	−0.485	0.032	14.963	0.308	[−0.546, −0.419]	1.000	< 0.001	Yes
H15	SS → INS	−0.074	0.047	1.566	0.005	[−0.166, 0.018]	1.000	0.118	No
Model fit				*Q^2^*	*R^2^*				
SRMR	0.037		AITR	0.152	0.563				

PE, Perceived ease of use; PU, Perceived usefulness; OPT, Optimism; INN, Innovativeness; DIS, Discomfort; INS, Insecurity; SS, School support; AITR, AI teaching readiness; Std. Dev., Standard Deviation; VIF, Variance Inflation Factor.

**Figure 2:**
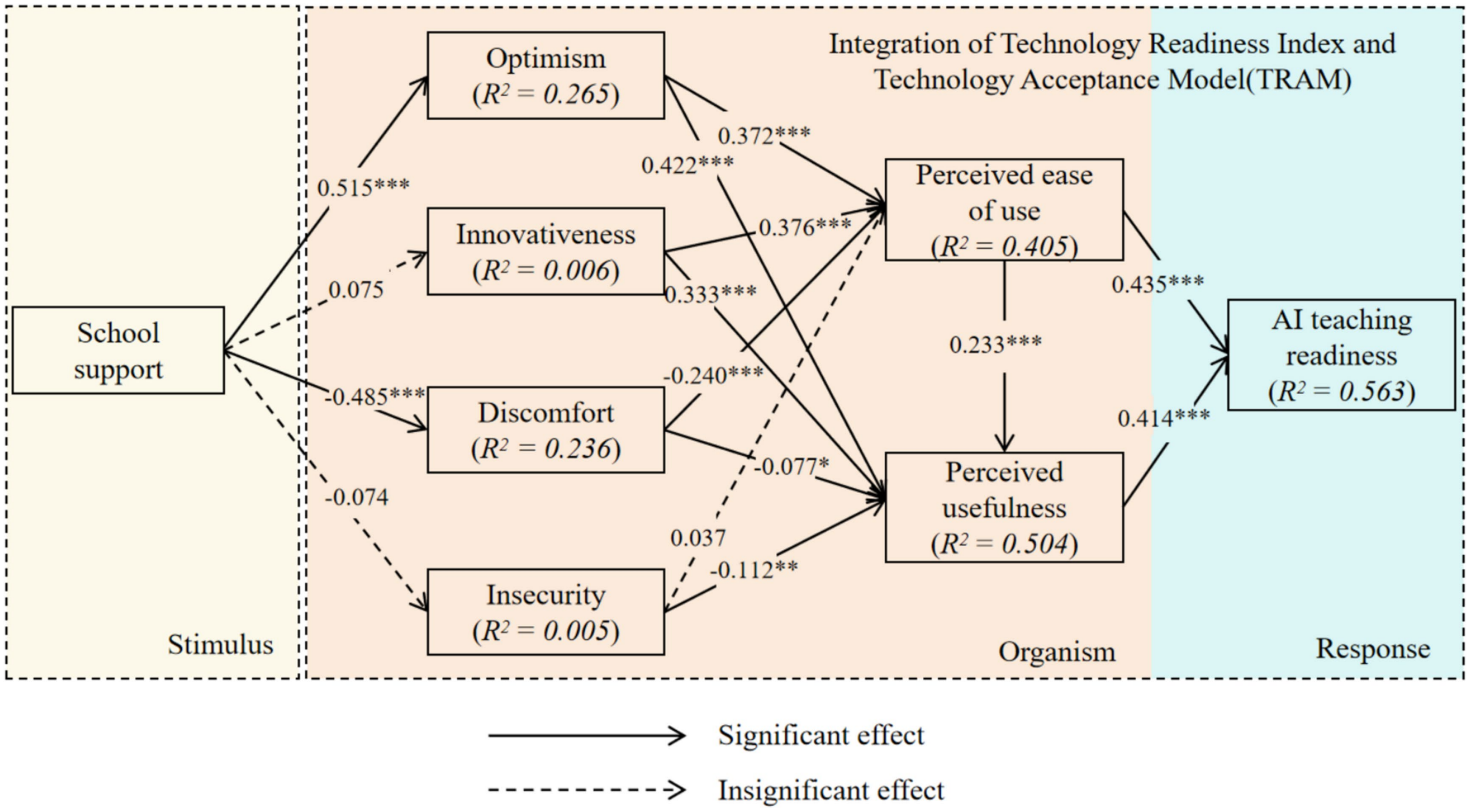
Results of path analysis. *p < 0.05, **p < 0.01 and ***p < 0.001.

Based on this assessment, this study tested the hypothesized relationships in the theoretical framework using a bootstrapping procedure with 5,000 resamples. As shown in Table 4 and Figure 2, perceived ease of use (*β = 0.435, t = 13.308, p < 0.001*) and perceived usefulness (*β = 0.414, t = 13.435, p < 0.001*) had positive effects on preservice STEM teachers’ AI teaching readiness (AITR), supporting H1 and H2. Optimism (*β = 0.372, t = 10.820, p < 0.001*) and innovativeness (*β = 0.376, t = 11.636, p < 0.001*) had positive effects on perceived ease of use, supporting H4 and H6. Discomfort (*β = −0.240, t = 6.960, p < 0.001*) had a significant negative effect on perceived ease of use, supporting H8. Perceived ease of use (*β = 0.233, t = 6.009, p < 0.001*) had a positive effect on perceived usefulness, supporting H3. By contrast, the relationship between insecurity and perceived ease of use (H10) was not significant (*β = 0.037, t = 1.076, p > 0.05*), indicating that insecurity did not significantly affect perceived ease of use. Optimism (*β = 0.422, t = 12.718, p < 0.001*) and innovativeness (*β = 0.333, t = 10.003, p < 0.001*) had positive effects on perceived usefulness, supporting H5 and H7. Discomfort (*β = −0.077, t = 2.303, p < 0.05*) and insecurity (*β = −0.112, t = 3.477, p < 0.01*) had significant negative effects on perceived usefulness, supporting H9 and H11. School support had a positive effect on optimism (*β = 0.515, t = 15.792, p < 0.001*), supporting H12. School support also had a significant negative effect on discomfort (*β = −0.485, t = 14.963, p < 0.001*), supporting H14. However, the effect of school support on innovativeness (H13) was not significant (*β = 0.075, t = 1.762, p > 0.05*), nor was the effect of school support on insecurity (H15) (*β = −0.074, t = 1.566, p > 0.05*). These results indicate that school support did not significantly influence innovativeness or insecurity.

Overall, most of the hypotheses in the proposed model were supported, including H1, H2, H3, H4, H5, H6, H7, H8, H9, H11, H12, and H14, whereas H10, H13, and H15 were not supported.

A bootstrapping procedure with 5,000 random resamples was further used to systematically examine the main chain-mediating paths in the theoretical model, so as to reveal the internal mechanisms through which the variables influence preservice STEM teachers’ AI teaching readiness. As shown in Table 5, the 95% confidence intervals for the chain-mediating paths SS → DIS → PU → AITR, SS → OPT → PU → AITR, SS → DIS → PEU → PU → AITR, SS → DIS → PEU → AITR, SS → OPT → PEU → AITR, and SS → OPT → PEU → PU → AITR did not include zero, indicating that the mediating effects were significant (Preacher and Hayes, 2008). These results suggest that optimism, discomfort, perceived ease of use, and perceived usefulness played significant chain-mediating roles between school support and AI teaching readiness.

**Table 5:** Results of mediation effect.

Paths	*β*	Std. dev.	*t*-value	Confidence interval	*p*-value
SS → DIS → PU → AITR	0.015	0.007	2.205	[0.003, 0.029]	0.027
SS → INS → PU → AITR	0.003	0.002	1.387	[−0.001, 0.009]	0.165
SS → INN→PU → AITR	0.010	0.006	1.694	[−0.001, 0.023]	0.090
SS → OPT→PU → AITR	0.090	0.012	7.386	[0.067, 0.114]	< 0.001
SS → INS → PEU → PU → AITR	0.000	0.000	0.752	[−0.001, 0.000]	0.452
SS → DIS → PEU → PU → AITR	0.011	0.003	3.973	[0.006, 0.017]	< 0.001
SS → INN→PEU → AITR	0.012	0.007	1.694	[−0.001, 0.027]	0.090
SS → DIS → PEU → AITR	0.051	0.009	5.341	[0.033, 0.071]	< 0.001
SS → OPT→PEU → AITR	0.083	0.012	6.996	[0.061, 0.108]	< 0.001
SS → INS → PEU → AITR	−0.001	0.002	0.773	[−0.005, 0.001]	0.439
SS → OPT→PEU → PU → AITR	0.019	0.004	4.514	[0.011, 0.027]	< 0.001
SS → INN→PEU → PU → AITR	0.003	0.002	1.626	[0.000, 0.006]	0.104

PEU, Perceived ease of use; PU, Perceived usefulness; OPT, Optimism; INN, Innovativeness; DIS, Discomfort; INS, Insecurity; SS, School support; AITR, AI teaching readiness; Std. dev., Standard Deviation.

The coefficient of determination (*R^2^*) was used to measure the extent to which the model explained the variance in the endogenous latent variables. In social science research, *R^2^* values of less than 0.10, between 0.10 and 0.50, and greater than 0.50 are generally interpreted as indicating low, moderate, and high explanatory power, respectively (Ozili, 2023). As shown in Figure 2, AI teaching readiness among preservice STEM teachers (AITR) had the highest *R^2^* value (*R^2^ = 0.563*), indicating that the model had strong explanatory power for this variable. This was followed by perceived usefulness (*R^2^ = 0.504*), whose explanatory power also reached a high level. The *R^2^* values of the remaining endogenous variables ranged from low to moderate, but all reached statistical significance and thus demonstrated a certain degree of explanatory power. Effect size (*f^2^*) was used to assess the magnitude of the explanatory contribution of exogenous latent variables to endogenous latent variables. Following Cohen’s (2009) criteria, *f^2^* values were interpreted as small (0.02), medium (0.15), and large (0.35) effects. The specific effect size results are presented in Table 5. *Q^2^* was used as an indicator of the model’s predictive relevance. In this study, the blindfolding algorithm produced a *Q^2^* value of 0.152 for AITR, which was above the threshold of 0.15, indicating that the model had good predictive relevance (Hair et al., 2011). In addition, this study evaluated model fit using the standardized root mean square residual (SRMR). The results showed that the SRMR value was 0.037, which was below the upper limit of 0.08 proposed by Hu and Bentler (1999), indicating good overall model fit. Meanwhile, the normed fit index (NFI) was 0.903, exceeding the criterion of 0.90 suggested by Bentler and Bonett (1980), further confirming the satisfactory fit of the model.

### 4.2 Asymmetric analysis

This study integrated two different analytical approaches: the symmetric approach of partial least squares structural equation modeling (PLS-SEM) and the asymmetric approach of fuzzy-set qualitative comparative analysis (fsQCA). PLS-SEM was mainly used to examine the linear relationships among variables and their significance, whereas fsQCA focuses on the necessary and sufficient conditions leading to a specific outcome and identifies multiple configurational pathways that can produce the same outcome. Unlike PLS-SEM, which focuses on the net effects of individual variables, fsQCA takes configurations of conditions as the unit of analysis and helps reveal asymmetric causal relationships in complex phenomena (Fiss, 2011).

#### 4.2.1 Analysis of necessity and sufficiency

This study used fsQCA 4.1 to conduct necessity and sufficiency analyses. In QCA, if a condition is always present when the outcome occurs, that condition can be regarded as a necessary condition for the outcome. Consistency is an important criterion for identifying necessary conditions. When the consistency value exceeds 0.90, the condition can be considered necessary for the outcome (Ragin, 2008a). The results, as shown in Table 6, indicated that the consistency values of all influencing factors were below 0.90, suggesting that there was no necessary condition influencing preservice STEM teachers’ AI teaching readiness. Therefore, it was necessary to further conduct configurational path analysis to reveal the interactions among variables and the multiple driving mechanisms underlying AI teaching readiness.

**Table 6:** Necessity analysis for high and low AI teaching readiness.

Causal conditions	AI teaching readiness		~AI teaching readiness	
	Consistency	Coverage	Consistency	Coverage
School support	0.73	0.75	0.58	0.58
~School support	0.59	0.59	0.75	0.73
Optimism	0.76	0.77	0.57	0.56
~Optimism	0.57	0.57	0.77	0.76
Innovativeness	0.76	0.74	0.59	0.57
~Innovativeness	0.56	0.58	0.73	0.75
Discomfort	0.61	0.59	0.75	0.71
~Discomfort	0.71	0.74	0.57	0.59
Insecurity	0.66	0.67	0.62	0.62
~Insecurity	0.63	0.63	0.67	0.66
Perceived ease of use	0.80	0.81	0.54	0.53
~Perceived ease of use	0.54	0.54	0.81	0.80
Perceived usefulness	0.79	0.80	0.53	0.53
~Perceived usefulness	0.54	0.54	0.80	0.79

~ indicates that the variable is at a low level.

In fsQCA, the frequency threshold should be adjusted according to the sample size. For small- to medium-sized samples, a threshold of 1 is commonly used, whereas larger samples require a higher threshold (Fiss, 2011; Ragin, 2008b). Therefore, based on the sample size and the distribution characteristics of the truth table, this study set the consistency threshold at 0.80 and the frequency threshold at 5. The software generated three types of solutions: complex, parsimonious, and intermediate solutions. This study reported the results mainly based on the intermediate solution, with the parsimonious solution used as a supplementary reference. The results are presented in Table 7.

**Table 7:** Configurations that lead to high AI teaching readiness.

Causal conditions	Solutions for high AI teaching readiness				
	C1	C2	C3	C4	C5
School support	⦁	●			⊗
Optimism	⦁		●	●	
Innovativeness		●	●	●	⦁
Discomfort	⊗		⊗		⊗
Insecurity		⊗		⦁	⦁
Perceived ease of use	●	●	●	●	●
Perceived usefulness	●	●	●	●	●
Raw coverage	0.43	0.35	0.41	0.39	0.27
Number of cases	73	40	63	59	18
Unique coverage	0.06	0.04	0.01	0.04	0.01
Consistency	0.95	0.96	0.96	0.95	0.96
Overall coverage	0.59				
Overall number of cases	253				
Overall consistency	0.93				

Large circles represent core conditions; small circles represent contributing conditions; ● Represents the presence of a condition; ⊗ Represents the absence of a condition; blank spaces represent that a condition may be either present or absent.

Table 7 presents the configurational pathways leading to high AI teaching readiness among preservice STEM teachers. The results showed an overall consistency of 0.93, indicating that these combinations of conditions consistently led to high AI teaching readiness (Ragin, 2006). The overall coverage was 0.59, suggesting that these configurations covered approximately 59% of the cases with high AI teaching readiness and thus had good explanatory power (Leppänen et al., 2021). Based on the similarity of core conditions, this study further classified these configurations into three patterns of high AI teaching readiness.

Pattern 1 (C1, C5): In this pattern, the core conditions promoting preservice STEM teachers’ AI teaching readiness were high perceived usefulness, high perceived ease of use, and low discomfort. This pattern included two structurally similar configurational pathways. Configuration C1 included, in addition to the core conditions, high school support and high optimism as peripheral conditions. This configuration explained 28.9% of the cases with high AI teaching readiness, making it the most frequent and most explanatory pathway among the five configurations. Configuration C5 included, in addition to the core conditions, high innovativeness, high insecurity, and low school support as peripheral conditions. This pathway explained 7.1% of the high-readiness cases.

Pattern 2 (C2): This pattern indicates that high school support, high innovativeness, high perceived usefulness, high perceived ease of use, and low insecurity jointly constitute important core conditions for enhancing AI teaching readiness among preservice STEM teachers. Support from the school environment and teachers’ individual traits reinforce each other, together forming an important pathway that promotes AI teaching readiness. This configuration explained 15.8% of the cases with high AI teaching readiness.

Pattern 3 (C3, C4): This pattern shows that the core conditions driving preservice STEM teachers to develop high AI teaching readiness include high optimism, high innovativeness, high perceived usefulness, and high perceived ease of use. This pattern included two similar configurational pathways. Configuration C3 included low discomfort as an additional core condition on the basis of the above four core conditions. Together with the other core conditions, this factor explained 24.9% of the high-readiness cases. Configuration C4 included high insecurity as a peripheral condition in addition to the four core conditions. This pathway explained 23.3% of the high-readiness cases.

#### 4.2.2 Robustness test

In fsQCA research, robustness testing is an important step for verifying the reliability and stability of the findings. Following the recommendations of Greckhamer et al. (2018), this study conducted robustness tests by changing the case frequency threshold and the consistency threshold. Specifically, the frequency threshold was reduced from 5 to 4. The results showed that the newly derived configurational pathways differed from the original results in only one factor combination, while the consistency and coverage indicators changed only slightly. Meanwhile, when the consistency threshold was increased from 0.80 to 0.90, the resulting configurational pathways remained completely consistent. Therefore, the configurational analysis results of this study demonstrate strong robustness.

## 5 Discussion

### 5.1 Findings of symmetric approach (PLS-SEM results)

The PLS-SEM results showed that the two core cognitive variables of the TAM, namely perceived ease of use and perceived usefulness, both had significant positive effects on AI teaching readiness. This finding is consistent with the theoretical expectations of TAM and is also supported by previous empirical studies. For example, Al-Adwan et al. (2024) found that both factors significantly influenced teachers’ continuance intention to use technology, while Almogren and Aljammaz (2022) reported similar findings in the mobile learning context. It indicates that when preservice teachers perceive AI tools as easy to use and pedagogically valuable, they are more likely to develop a higher level of AI teaching readiness. In addition, perceived ease of use had a significant positive effect on perceived usefulness, which is consistent with the findings of Lv et al. (2025) and Xia et al. (2025). This suggests that a positive operational experience helps strengthen preservice teachers’ judgments of the pedagogical value of AI teaching tools and further promotes the formation of their teaching readiness.

This study examined the influence paths of the four core dimensions of technology readiness on the core variables of TAM from the perspective of the TRAM model. Specifically, positive technology traits, namely optimism and innovativeness, had significant positive effects on both perceived ease of use and perceived usefulness. This indicates that when preservice STEM teachers hold positive attitudes toward AI technologies and have a stronger willingness to try new technologies, they are more likely to perceive the pedagogical value of AI tools (PU) and to regard these technologies as easy to master (PEU). This finding is consistent with the basic assumption of TR theory that positive dimensions play a facilitating role, and it also aligns with the findings of Jin (2019) and Anh et al. (2024). Meanwhile, the effects of negative technology traits, namely discomfort and insecurity, on TAM variables showed certain differences. Discomfort had significant inhibitory effects on both perceived usefulness and perceived ease of use, suggesting that when teachers experience anxiety due to technological complexity or operational pressure, they are less likely to recognize the potential value of AI and less likely to form expectations of smooth and easy use (Venkatesh, 2000; Xia et al., 2025). However, insecurity had a significant negative effect only on perceived usefulness, while its effect on perceived ease of use was not significant. This may indicate that insecurity is more closely related to teachers’ concerns about the reliability, potential risks, and consequences of using AI technologies; therefore, it mainly affects their judgments of technological value rather than their perceptions of operational difficulty. This interpretation is generally consistent with the definition of insecurity in technology readiness theory (Parasuraman, 2000). Nevertheless, this explanation remains an inference based on the findings of this study and theoretical logic, and should be further verified in future research.

Meanwhile, this study found that school support had differentiated effects on the four dimensions of technology readiness (TR). First, school support had a significant positive effect on optimism, which is consistent with the findings of Shen et al. (2024). This indicates that when schools provide sufficient support in terms of hardware and software conditions, curriculum resources, training opportunities, and technical services, preservice STEM teachers are more likely to develop positive expectations about the pedagogical potential of AI technologies. Second, school support had a significant negative effect on discomfort, which is consistent with the findings of Solís et al. (2023). This suggests that organizational support can reduce teachers’ discomfort and operational pressure caused by technological complexity or unfamiliarity through training demonstrations, peer collaboration, and technical guidance. However, the effects of school support on innovativeness and insecurity were not significant, which was inconsistent with the research hypotheses. This result may be related to the different psychological attributes of the dimensions of technology readiness. Innovativeness mainly reflects individuals’ tendency to actively try new technologies and act as technology pioneers. It may be more strongly influenced by intrinsic motivation, personality traits, and long-term professional development experiences, and may not necessarily be directly enhanced in the short term through general school support (Parasuraman and Colby, 2014; Ranihusna et al., 2021). In addition, preservice teachers are still in the process of professional learning and competence development, and their technological innovativeness and teaching practice experience may not yet be fully developed. By contrast, insecurity more strongly reflects individuals’ concerns about technological reliability, data privacy, ethical risks, and the consequences of technology use. Such risk judgments often involve values, professional ethics, and broader understandings of the direction of technological development. Therefore, resource provision and routine training at the school level alone may be insufficient to directly eliminate preservice teachers’ concerns about the potential consequences of AI technologies (Akgun and Greenhow, 2021). It should be noted that the above explanation is mainly an inference based on technology readiness theory, the characteristics of the present sample, and related literature, and thus requires further verification among different teacher groups and AI teaching application contexts. Overall, these differentiated findings suggest that school support can more directly enhance preservice teachers’ positive expectations and alleviate operational pressure. However, further cultivating their technological innovativeness and reducing deeper risk concerns may require curriculum reform, practical experience, ethics education, and continuous professional development support.

This study further examined the chain-mediating effects through which school support influences AI teaching readiness via the dimensions of technology readiness and the core variables of TAM. The results showed that school support exerted positive chain-mediating effects through the path of “optimism → perceived usefulness/perceived ease of use → AI teaching readiness.” This indicates that when preservice STEM teachers perceive school support in terms of resources, training, and technical services, they are more likely to develop positive expectations about the pedagogical application of AI, which in turn enhances their perceptions of the usefulness and ease of use of AI tools and ultimately strengthens their AI teaching readiness. At the same time, school support can also promote AI teaching readiness by reducing discomfort and further influencing perceived usefulness and perceived ease of use. In other words, a higher level of school support helps alleviate teachers’ unease and pressure when using AI technologies, thereby improving their judgments of technological ease of use and pedagogical value. Furthermore, the triple chain-mediating paths involving “perceived ease of use → perceived usefulness” were also significant. This suggests that school support can not only influence ease-of-use perceptions by improving technology-related psychological experiences but also further strengthen usefulness judgments on the basis of enhanced ease of use, thereby progressively promoting AI teaching readiness. Overall, school support operates through multiple chain-mediating paths of “technology readiness → TAM cognitive variables → AI teaching readiness,” highlighting the importance of optimizing the school support environment, improving technology-related psychological experiences, and strengthening technology-related cognition in enhancing AI teaching readiness among preservice STEM teachers.

### 5.2 Findings of asymmetric approach (fsQCA results)

The fsQCA configurational analysis results showed that AI teaching readiness among preservice STEM teachers was characterized by clear multiple conjunctural causation. Perceived ease of use and perceived usefulness repeatedly appeared in multiple high-readiness configurations, indicating that the core cognitive variables of TAM remained important conditions for the formation of AI teaching readiness.

Pattern 1 (C1, C5) showed that high perceived usefulness, high perceived ease of use, and low discomfort jointly constituted an important pathway to high AI teaching readiness. This indicates that preservice teachers are more likely to develop higher readiness when they recognize the pedagogical value of AI, perceive it as easy to use, and experience less operational pressure. Among these configurations, C1 had the highest coverage and also included high school support and high optimism, suggesting that school support in terms of resources, training, and technical services can strengthen teachers’ positive technology-related expectations and thereby promote their AI teaching readiness (Molefi et al., 2024). By contrast, although C5 was characterized by low school support, high innovativeness appeared as a peripheral condition, indicating that individual innovativeness may, to some extent, compensate for insufficient external support.

Pattern 2 (C2) reflected the configurational feature of “high school support + high innovativeness + high perceived usefulness + high perceived ease of use + low insecurity.” This result indicates that when external support, individual innovativeness, and positive technology-related cognition are simultaneously present, and when teachers have relatively low concerns about the risks of AI technologies, they are more likely to develop a high level of AI teaching readiness.

Pattern 3 (C3, C4) highlighted the important roles of optimism and innovativeness as two positive dimensions of technology readiness, reflecting a readiness formation pattern that is more strongly driven by individual psychological traits. Even when school support is not a core condition, preservice teachers with higher levels of optimism and innovativeness may still develop higher AI teaching readiness because of their positive technology attitudes and tendency to actively explore new technologies. Notably, insecurity was still present in C4 and C5. However, high innovativeness and positive technology-related cognition may weaken its negative influence, enabling teachers to accept AI technologies even when certain risk concerns remain. This is generally consistent with the findings of Lyu et al. (2025).

Overall, the fsQCA results complement the findings of the symmetric PLS-SEM analysis. They indicate that there is no single pathway to high AI teaching readiness; rather, high readiness can be achieved through multiple pathways, including school-support reinforcement, cognitive-experience-driven pathways, and individual-trait-dominant pathways.

### 5.3 Comparison of PLS-SEM and fsQCA results

A comprehensive comparison of the PLS-SEM and fsQCA results shows that the two methods provide both consistent and complementary explanations of AI teaching readiness among preservice STEM teachers.

First, in the PLS-SEM results, perceived usefulness and perceived ease of use both had significant positive effects on AI teaching readiness and were the most important direct predictors. Similarly, in the fsQCA results, perceived usefulness and perceived ease of use repeatedly appeared as core conditions in the high-readiness configurations. This indicates that these two cognitive factors are key drivers of high AI teaching readiness among preservice STEM teachers, showing a high degree of consistency between the two methods.

Second, the PLS-SEM results showed that discomfort had significant negative effects on both PEU and PU. This finding was also echoed in the fsQCA results: the combination of “low discomfort + high perceived usefulness + high perceived ease of use” appeared repeatedly in Pattern 1 and Pattern 3. This suggests that reducing technology-related pressure and operational anxiety is an important psychological mechanism for enhancing AI teaching readiness. In this regard, the fsQCA results further complement the PLS-SEM findings by revealing contextual differences that linear analysis alone may not capture.

Finally, the PLS-SEM results showed that school support significantly influenced optimism and discomfort, but had no significant effects on innovativeness or insecurity. The fsQCA results further indicated that school support worked jointly with positive technology-related psychological traits and TAM-based cognitive variables in certain pathways. For example, in C1, high school support appeared together with high optimism, low discomfort, high perceived usefulness, and high perceived ease of use. In C2, high school support, high innovativeness, low insecurity, high perceived usefulness, and high perceived ease of use jointly formed a high-readiness configuration. In contrast, C5 showed that even when school support was low, high innovativeness and high perceived usefulness/perceived ease of use could still lead to high readiness. This indicates that fsQCA can reveal the complex mechanism of “multiple pathways to the same outcome,” thereby complementing the limitations of linear path analysis in PLS-SEM.

Overall, the two methods jointly indicate that school support as an external environment, technology readiness as individual psychological traits, and perceived usefulness and perceived ease of use as technology-related cognitive appraisals together constitute a multilevel mechanism influencing AI teaching readiness among preservice STEM teachers. PLS-SEM revealed the linear influence paths among the variables, whereas fsQCA further showed that high AI teaching readiness does not depend on a single causal pathway but can be achieved through multiple combinations of conditions.

## 6 Theoretical and practical implications

### 6.1 Theoretical implications

Drawing on an integrated perspective of technology readiness (TR), the technology acceptance model (TAM), and the stimulus–organism–response (SOR) framework, this study constructed a mechanism model of AI teaching readiness among preservice STEM teachers and analyzed it using both PLS-SEM and fsQCA. In doing so, this study extends theories of teachers’ technology adoption in the following ways.

First, this study extends the explanatory scope of TAM from traditional technology use intention to AI teaching readiness. The results showed that both perceived usefulness and perceived ease of use had significant positive effects on AI teaching readiness, and that perceived ease of use further promoted perceived usefulness. This indicates that, in AI teaching contexts, preservice teachers need not only to recognize the pedagogical value of AI tools but also to develop expectations that these tools are accessible and operationally feasible. This finding further confirms the applicability of TAM in the context of emerging educational technologies (Fussell and Truong, 2021; García et al., 2024) and adds empirical evidence from the population of preservice STEM teachers.

Second, this study refines the mechanism through which TR theory operates in AI teaching contexts. By introducing the four dimensions of optimism, innovativeness, discomfort, and insecurity, this study found that TR does not influence technology adoption in a single direction. Rather, different psychological traits exert differentiated effects on technology-related cognition. Specifically, optimism and innovativeness significantly promoted perceived usefulness and perceived ease of use, while discomfort significantly inhibited both types of technology-related cognition. In contrast, insecurity mainly affected perceived usefulness, whereas its effect on perceived ease of use was not significant. This finding suggests that TR variables not only influence use intention but also affect AI teaching readiness, a more antecedent psychological state, through cognitive appraisal pathways. In this way, the study extends the explanatory boundary of TR theory in educational technology contexts (Kaushik and Agrawal, 2021; Zhao et al., 2025).

Third, this study applies SOR theory to the context of AI teaching readiness among preservice STEM teachers and clarifies the psychological processing pathway of “environment–traits–cognition–readiness.” Specifically, school support was defined as the external stimulus (S), technology readiness and TAM-based cognitive variables were defined as internal organismic states (O), and AI teaching readiness was defined as the response (R). This framework not only explains how external support influences teachers’ AI teaching readiness but also further reveals that school support does not operate in a direct and linear manner. Instead, it exerts indirect effects through positive and negative technology-related psychological traits and technology-related cognitive appraisals. Therefore, the introduction of SOR establishes a clearer theoretical bridge between TR and TAM and expands the explanatory scope of AI technology adoption research in teacher education (Sun P. et al., 2025; Wang et al., 2025).

Fourth, by combining PLS-SEM and fsQCA, this study further reveals both the linear pathways and configurational effects underlying the formation of AI teaching readiness. The PLS-SEM results indicate that significant path relationships exist among school support, technology readiness, and TAM variables. The fsQCA results further show that there is no single pathway leading to high AI teaching readiness; rather, it can be achieved through multiple configurations, such as school-support reinforcement, cognitive-experience-driven pathways, and individual-trait-dominant pathways. This finding complements the limitations of traditional linear models and provides a causal complexity perspective for understanding AI teaching readiness among preservice STEM teachers.

Overall, this study does not simply combine TR, TAM, and SOR in an additive manner. Instead, it further clarifies their functional roles in the formation of AI teaching readiness: SOR provides the overarching explanatory framework, TR reveals individual differences in technology-related psychological traits, and TAM explains the mechanism of technology-related cognitive appraisal. Accordingly, this study offers a more systematic, hierarchical, and complexity-oriented theoretical framework for explaining AI teaching readiness among preservice STEM teachers.

### 6.2 Practical implications

The findings of this study have important implications for teacher education and school management practices. First, perceived usefulness and perceived ease of use are key factors in enhancing AI teaching readiness among preservice STEM teachers. Therefore, universities and teacher training institutions should strengthen the demonstration, operational guidance, and practical experience of AI teaching tools, so that preservice teachers can perceive the value and convenience of AI technologies in authentic teaching contexts. Second, this study found that positive technology readiness, such as optimism and innovativeness, plays a compensatory and facilitating role in certain pathways. This suggests that teacher education programs should not only cultivate technological skills but also stimulate preservice teachers’ innovative awareness and positive mindsets. Project-based learning, teaching innovation activities, and case sharing can be used to enhance their adaptability and confidence when facing new technologies (Meegan and Young, 2025; Yang et al., 2024; Lai et al., 2024; Almusawi and Durugbo, 2024). Third, school support plays an important role in reducing preservice teachers’ discomfort. Therefore, schools should continue to invest in technological equipment, technical services, professional training, and peer support mechanisms, and build a stable, accessible, and user-friendly technical support environment. This would enable preservice teachers to improve their familiarity with and sense of control over AI tools through continuous practice and timely feedback, thereby reducing the pressure and anxiety caused by technological complexity and operational uncertainty (Zhang and Cao, 2025; Shen et al., 2025).

In addition, regarding insecurity, although the PLS-SEM results showed that school support did not have a significant direct effect on insecurity, the fsQCA results indicated that low insecurity was an important condition in some configurations leading to high AI teaching readiness. Therefore, schools and educational institutions still need to provide institutional safeguards through improved technology ethics guidelines, data security training, and risk warning mechanisms, so as to reduce preservice teachers’ concerns about the reliability, ethical risks, and data security issues associated with AI technologies (Prainsack and Forgó, 2024; Zhang et al., 2024).

Finally, the significance of multiple chain-mediating paths suggests that school administrators and policymakers should design support strategies from an integrated “environment–psychology–cognition” perspective. By optimizing organizational conditions, improving technology-related psychological experiences, strengthening technology-related cognition, and responding to risk concerns, they can jointly promote a higher level of AI teaching readiness among preservice STEM teachers.

## 7 Limitations and future research

Although this study has certain innovations in theoretical integration and methodological application, several limitations remain. First, although the sample size met the basic requirements for PLS-SEM and fsQCA analyses, the sample was drawn only from preservice STEM teachers at two universities in western China. The relatively limited geographical coverage and types of institutions may restrict the generalizability of the findings to other regions, different types of universities, and other teacher groups. Therefore, future research could expand the sample sources by including preservice teachers from eastern, central, and western regions of China, as well as from different types of institutions, such as normal universities and comprehensive universities. Cross-regional, cross-institutional, and cross-disciplinary comparative studies could also be conducted to improve the representativeness and external validity of the findings. Second, this study collected data through self-reported questionnaires, which may be subject to social desirability bias and individual subjective judgment. Future research could combine multiple sources of data, such as interviews, classroom observations, teaching practice performance, or learning analytics data, to enhance the robustness of the findings. Meanwhile, this study mainly used cross-sectional data to examine the relationships among school support, technology readiness, technology acceptance perceptions, and AI teaching readiness. However, preservice teachers’ technology-related psychological traits and cognitive appraisals may dynamically evolve with learning experiences, practice opportunities, and changes in school contexts. Therefore, future studies could adopt longitudinal or experimental designs to further reveal the temporal effects and causal pathways underlying the formation mechanism of AI teaching readiness.

Finally, regional differences in digital education development, school resource allocation, AI education policy support, and teacher education models may influence the relationships among the variables in the proposed model. Future research could further introduce moderating variables such as regional differences, institutional resources, technological infrastructure, and AI training opportunities to examine the boundary conditions of the relationships among school support, technology readiness, technology acceptance perceptions, and AI teaching readiness, thereby further improving the explanatory power of the model.

## Data Availability

The original contributions presented in the study are included in the article. Further inquiries can be directed to the corresponding author.
